# Unilateral testicular seminoma with simultaneous contralateral torsion: a case report

**DOI:** 10.1186/1752-1947-6-199

**Published:** 2012-07-16

**Authors:** Kazumi Taguchil, Takahiro Yasui, Taku Naiki, Yukihiro Umemoto, Yoshiyuki Kojima, Noriyasu Kawai, Keiichi Tozawa, Yutaro Hayashi, Kenjiro Kohri

**Affiliations:** 1 Department of Nephro-urology, Nagoya City University Graduate School of Medical Sciences, Kawasumi, Mizuho-cho, Mizuho-ku, Nagoya, 467-8601, Japan

## Abstract

**Introduction:**

Testicular germ cell tumors are the most common malignancies in men. Testicular torsion is also a scrotal phenomenon seen in adolescence and adulthood. The co-occurrence of these two scrotal disorders is extremely rare.

**Case presentation:**

A 28-year-old East Asian man presented at our hospital with painless bilateral scrotal swelling. Both scrotal ultrasonography and computed tomography findings showed bilateral testicular tumors, and magnetic resonance imaging demonstrated a lack of enhancement in his right testis, indicating a hemorrhagic infarction and a left testicular tumor. After a bilateral orchiectomy, the intraoperative and histopathological findings revealed a left seminoma with a complicating contralateral testicular torsion that had developed with hemorrhagic infarction.

**Conclusion:**

Testicular germ cell tumor with contralateral torsion is extremely rare. We could differentiate this case from bilateral testicular tumors appropriately using magnetic resonance imaging, and suggest that magnetic resonance imaging examination may be necessary to diagnose bilateral testicular masses.

## Introduction

Testicular germ cell tumors (TGCTs) are the most common malignancies in men, including young adults, and their incidence has been increasing in almost all developed countries [1,2]. Testicular torsion is also a scrotal phenomenon seen not only in adolescence, but also in adulthood [3]. These two scrotal disorders can complicate each other, but cases in which each testis has each scrotal disease independently are extremely rare [4]. In this report, we encountered a 28-year-old man presenting with a simultaneous seminoma and contralateral torsion. The scrotal disorders seemed to suggest bilateral TGCTs as a differential diagnosis, but corrective treatment was possible with the aid of magnetic resonance imaging (MRI).

## Case presentation

**Figure 1:**
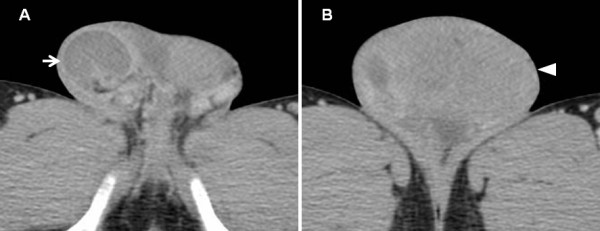
**Contrast-enhanced computed tomography. (A)** The right testis was normal in size with decreased enhancement (arrow). **(B)** The left testis was enlarged with heterogeneous enhancement (arrowhead).

A healthy, engaged 28-year-old East Asian man presented at our hospital with a two-week history of right-side scrotal swelling and a three-month history of left-side scrotal enlargement. He had complained of temporary dull pain in his right scrotum, but the pain had disappeared within a day. He had no history of prior scrotal surgery, cryptorchidism or infections. On physical examination, his right scrotum was slightly swollen, whereas his left scrotum had marked swelling and a hard mass was detectable. A scrotal ultrasound showed heterogeneous bilateral scrotal masses that were hypoechoic on the right but hyperechoic on the left. Doppler ultrasonography indicated a diffuse hypovascular area in the right scrotal mass but iso- and hypervascular on the left. Pelvic contrast-enhanced computed tomography (CT) demonstrated that his right testis was normal in size with decreased enhancement, whereas his left testis was much larger with heterogeneous enhancement (Figure 1).

**Figure 2:**
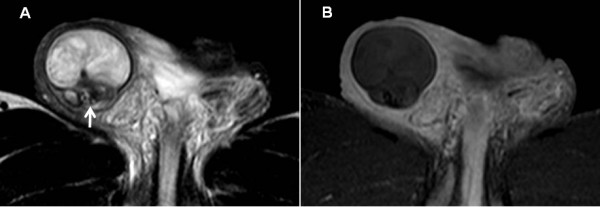
**Magnetic resonance imaging of the right testis. (A)** T2-weighed image showing that intratesticular intensity was slightly high and a serpiginous vessel (arrow). **(B)** Dynamic contrast-enhanced subtraction, showing that the intratesticular space had a lack of enhancement.

**Figure 3:**
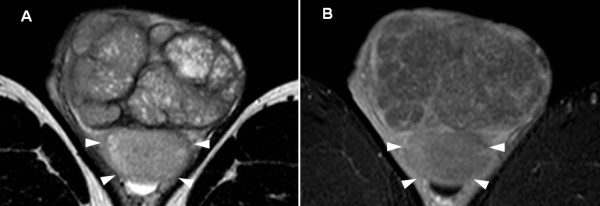
**Magnetic resonance imaging of the left testis. (A)** T2-weighed image showing a large multinodular tumor with heterogeneous high intensity. **(B)** Dynamic contrast-enhanced subtraction showing that the left testis had only slight enhancement. There seemed to be a normal left testis (arrowheads), although the border between the tumor and normal testis was not very clear.

These findings seemed to suggest bilateral TGCTs as a differential diagnosis, but his right testis did not have typical features of a TGCT; therefore, we examined his bilateral testes with MRI. His right testis had a serpiginous vessel and the intratesticular lesion showed high intensity on the T2-weighted image (Figure 2A), but lack of contrast enhancement (Figure 2B). His left testis had a large multinodular tumor with high intensity on the T2-weighted image (Figure 3). According to the MRI findings, his right and left testes were diagnosed with hemorrhagic infarction and a testicular tumor, respectively. Before further treatment, we performed a sperm test. Sperm were present (8 million/mL) and were cryopreserved for use in an assisted reproductive technique, although his levels of luteotropic hormone (15.6mIU/mL) and follicle-stimulating hormone (49.0mIU/mL) were high.

Based on the preoperative diagnosis, a transverse scrotal incision was made to allow right scrotal exploration. Since inspection of his right testis revealed a necrotic testis with 270° intravaginal torsion of the spermatic cord, his right testis was removed. Left radical orchiectomy was then carried out.

**Figure 4:**
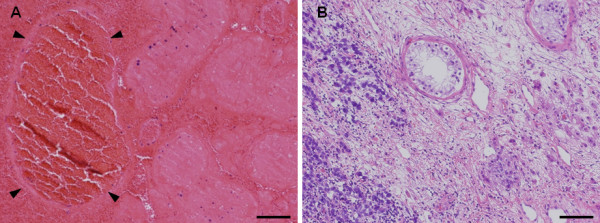
**Hematoxylin-eosin staining of both sides of the testis. (A)** Hematoxylin-eosin staining demonstrated hemorrhagic infarction with markedly dilated vessels (arrowheads) in the right testis. Bar = 50μm. **(B)** Hematoxylin-eosin staining demonstrated infiltration of seminoma cells with atrophy of seminiferous tubules in the left testis. Bar = 50μm.

Pathological examination demonstrated hemorrhagic infarction and dilated vessels in his right testis (Figure 4A), and typical seminoma cells and atrophy of seminiferous tubules in his left testis (Figure 4B). Preoperative laboratory examination showed a high lactic dehydrogenase level of 580IU/L and normal alpha-fetoprotein and human chorionic gonadotropin levels in his serum. Because an abdominal CT showed para-aortic retroperitoneal lymphadenopathy, 9mm in size, we diagnosed seminoma stage IIA, which has a good prognosis according to the definition of the International Germ Cell Cancer Consensus Group. Therefore, our patient was promptly given chemotherapy in three cycles of bleomycin, etoposide and cisplatin [5]. At the end of the three cycles, a follow-up CT scan showed complete resolution of the previous retroperitoneal lymphadenopathy and the tumor marker level was undetectable. After bilateral castration his serum level of testosterone was markedly decreased, so we started supplemental therapy of testosterone for his erectile function.

## Discussion

The estimated incidence of testicular torsion in men is 4.5 per 100,000 [4], and the incidence of TGCT in men during their lifetime is 5.1 per 100,000 [6]. TGCT with contralateral testicular torsion is still extremely rare. There have been some case reports of TGCT with ipsilateral testicular torsion, most detected after presenting with acute scrotal pain as a symptom of torsion. The causes of TGCT with developed torsion remain unclear, but there is a report that 96% of patients with testicular torsion have anatomical variations, such as bell clapper deformity and a long mesorchium [7]. Srinivasan *et al.* reported that hyperactivity of the cremasteric reflex, stimulated by cold weather, has been postulated as the physiopathology of acute testicular torsion [8].

In general, the differential diagnosis to distinguish from bilateral TGCT is important because the intraoperative approaches, such as ligation of the spermatic cord and the site of incision, are significantly different depending on whether the testis is cancerous. In fact, the incidence of bilateral TGCT in patients with a TGCT has been reported as 1 to 3% [9], and patients with seminoma were especially at significantly higher risk for bilateral disease than those with a nonseminomatous germ cell tumor. In our patient, his right testis was diagnosed with hemorrhagic infarction with torsion by MRI, intraoperative and histopathological findings. Microscopic examination revealed markedly dilated vessels caused by torsion. These findings proved the diagnosis as a testicular infarction, not a tumor.

Most solid intratesticular masses should be considered malignant, and radical orchiectomy is the treatment of choice. To avoid unnecessary orchiectomy, however, it is extremely important to recognize the imaging features of various benign intratesticular mass lesions, including orchitis, hemorrhage, ischemia and infarction, fibrosis, and dilatation of the rete testis. Cohen *et al.* reported the first case of a testicular mixed germ cell tumor with contralateral testicular torsion [10]. Although they mainly used a radionuclide scrotal scan to diagnose the torsion, it would not be useful for other scrotal disorders. Although Doppler ultrasonography and contrast-enhanced CT imaging are often used as diagnostic tools for acute scrotal disease, including torsion, inconclusive results may often be observed and their efficacy depends on the operator’s skill [11]. Dynamic contrast-enhanced subtraction MRI could provide information about testicular perfusion and is useful to diagnose scrotal disorders [12]. Diffusion-weighted MRI can also enable the detection of testicular torsion [11]. Tsili *et al.* indicated that the sensitivity, specificity and accuracy of MRI in the diagnosis of malignant testicular tumors were 100%, 87.5% and 96.4%, respectively [13]. The advantages of this technique include the acquisition of precise anatomic information, satisfactory tissue contrast, and imaging in various planes. MRI is an efficient diagnostic tool for the evaluation of testicular masses, especially in preoperative differentiation, as in our case. In addition, it is important to preserve postoperative fertility in patients with both unilateral and bilateral TGCTs. The most effective means of preserving fertility in these patients is sperm cryopreservation before the initiation of cancer-directed therapy [14]. In this case, we could preserve his sperm before orchiectomy and avoid a hasty operation with useful findings from MRI.

## Conclusions

This is an extremely rare report of a patient presenting with simultaneous right testicular torsion and left seminoma, which could be differentiated from bilateral TGCT by MRI. We believe that accurate examination using MRI is necessary to diagnose bilateral scrotal masses and evaluate the perioperative conditions to ensure the postoperative quality of life of patients.

## Consent

Written informed consent was obtained from the patient for publication of this case report and any accompanying images. A copy of the written consent is available for review by the Editor-in-Chief of this journal.

## Competing interests

The authors declare that they have no competing interests.

## Authors’ contributions

KTa drafted the report and approved the final version of the manuscript. TN carried out the immunoassays and approved the final version of the manuscript. YU, YK, NK, KTo, YH and KK cared for the patient and approved the final version of the manuscript. TY drafted the report, cared for the patient and approved the final version of the manuscript. All authors read and approved the final manuscript.

## References

[B1] Huyghe E Matsuda T Thonneau P Increasing incidence of testicular cancer worldwide: a review J Urol 2003 170 5 11 10.1097/01.ju.0000053866.68623.da 12796635

[B2] Richardson LC Wingo PA Zack MW Zahran HS King JB Health-related quality of life in cancer survivors between ages 20 and 64 years Cancer 2008 112 1380 1389 10.1002/cncr.23291 18219664

[B3] Cummings JM Boullier JA Sekhon D Bose K Adult testicular torsion J Urol 2002 167 2109 2110 10.1016/S0022-5347(05)65096-3 11956451

[B4] Mansbach JM Forbes P Peters C Testicular torsion and risk factors for orchiectomy Arch Pediatr Adolesc Med 2005 159 1167 1171 10.1001/archpedi.159.12.1167 16330742

[B5] Garcia-del-Muro X Maroto P Gumà J Sastre J López Brea M Arranz JA Lainez N Soto de Prado D Aparicio J Piulats JM Pérez X Germá-Lluch JR Chemotherapy as an alternative to radiotherapy in the treatment of stage IIA and IIB testicular seminoma: a spanish germ cell cancer group study J Clin Oncol 2008 26 5416 5421 10.1200/JCO.2007.15.9103 18936476

[B6] Townsend JS Richardson LC German RR Incidence of testicular cancer in the United States, 1999–2004 Am J Mens Health 2010 4 353 360 10.1177/1557988309356101 20031937

[B7] Favorito LA Cavalcante AG Costa WS Anatomic aspects of epididymis and tunica vaginalis in patients with testicular torsion Int Braz J Urol 2004 30 420 424 15610580 10.1590/s1677-55382004000500014

[B8] Srinivasan AK Freyle J Gitlin JS Palmer LS Climatic conditions and the risk of testicular torsion in adolescent males J Urol 2007 178 2585 2588 10.1016/j.juro.2007.08.049 17945301

[B9] Bulent A Taner DR Tolga T Sertac Y Celik T Ferruh Z Haluk O Bilateral testicular germ cell tumors in Turkey: increase in incidence in last decade and evaluation of risk factors in 30 patients J Urol 2007 178 129 133 10.1016/j.juro.2007.03.027 17499297

[B10] Cohen M Sova Y Grunwald I Resnick M Stein A A rare simultaneous presentation of testicular mixed germ cell tumor with a contralateral testis torsion Urology 2000 55 590ix 590xi 10.1016/s0090-4295(99)00586-5 10754182

[B11] Maki D Watanabe Y Nagayama M Ishimori T Okumura A Amoh Y Nakashita S Terai A Dodo Y Diffusion-weighted magnetic resonance imaging in the detection of testicular torsion: feasibility study J Magn Reson Imaging 2011 34 1137 1142 10.1002/jmri.22698 21928380

[B12] Watanabe Y Dohke M Ohkubo K Ishimori T Amoh Y Okumura A Oda K Hayashi T Dodo Y Arai Y Scrotal disorders: evaluation of testicular enhancement patterns at dynamic contrast-enhanced subtraction MR imaging Radiology 2000 217 219 227 11012448 10.1148/radiology.217.1.r00oc41219

[B13] Tsili AC Argyropoulou MI Giannakis D Sofikitis N Tsampoulas K MRI in the characterization and local staging of testicular neoplasms AJR Am J Roentgenol 2010 194 682 689 10.2214/AJR.09.3256 20173145

[B14] Levine J Canada A Stern CJ Fertility preservation in adolescents and young adults with cancer J Clin Oncol 2010 28 4831 4841 10.1200/JCO.2009.22.8312 20458029

